# The CC Genotype of the Delta-Sarcoglycan Gene Polymorphism rs13170573 Is Associated with Obstructive Sleep Apnea in the Chinese Population

**DOI:** 10.1371/journal.pone.0114160

**Published:** 2014-12-04

**Authors:** Rensong Ye, Wenlan Yang, Yiming Yuan, Xingqi Deng

**Affiliations:** 1 Department of Internal Medicine, The Affiliated Shanghai Eighth People’s Hospital of Jiangsu University, Shanghai, China; 2 Department of Emergency Medicine, The Center Hospital of Minhang District, Shanghai, China; 3 Department of Respiratory Medicine, The Affiliated Shanghai Pulmonary Hospital of Tongji University, Shanghai, China; 4 Department of Gerontology, The Affiliated West China Hospital of Sichuan University, Chengdu, China; National Institute for Viral Disease Control and Prevention, CDC, China, China

## Abstract

Obstructive sleep apnea (OSA) is a highly heterogeneous sleep disorder, and increasing evidence suggests that genetic factors play a role in the etiology of OSA. Airway muscle dysfunction might promote pharyngeal collapsibility, mutations or single nucleotide polymorphisms (SNPs) in the delta-sarcoglycan (SCGD) gene associated with muscle dysfunction. To evaluate if SCGD gene SNPs are associated with OSA, 101 individuals without OSA and 97 OSA patients were recruited randomly. The genotype distributions of SNPs (rs157350, rs7715464, rs32076, rs13170573 and rs1835919) in case and control populations were evaluated. The GG, GC and CC genotypes of rs13170573 in control and OSA groups were 51.5% and 37.1%, 36.6% and 35.1%, and 11.9% and 27.8%, respectively. Significantly fewer OSA patients possessed the GG genotype and significantly more possessed the CC genotype compared with controls. Further multivariate logistic regression analysis showed that the CC genotype was an independent risk factor for OSA, with an odds ratio (OR) of 2.17 (95% confidence interval [CI]: 1.19–6.01). Other factors, such as age ≥50 years, male gender, body mass index (BMI) ≥25 kg/m^2^, low-density lipoprotein cholesterol (LDL-C) level ≥3.33 mg/dL, smoking and hypertension, were also independent risk factors for OSA in our multivariate logistic regression model.

## Introduction

Sleep disorders are highly prevalent worldwide and have significant public health implications. Sleep disorders can be classified into four main categories: (1) dyssomnias, (2) parasomnias, (3) sleep disorders associated with mental, neurologic, or other medical disorders, and (4) proposed sleep disorders [1], [2]. Obstructive sleep apnea (OSA), one of the most prevalent sleep disorders, is a highly heterogeneous condition defined by recurrent reductions or cessations in breathing during sleep [2]. Clinical definitions of OSA are based on apnea (breathing cessation for >10 seconds), hypopnea (reduced respiratory airflow by 30% with a 4% decrease in oxygen saturation) and the apnea-hypopnea index (AHI; number of apnea and hypopnea events recorded per hour of sleep) [1]–[4]. Studies have demonstrated that OSA is associated with increased cardiovascular and cerebrovascular morbidity and mortality [2]. Increasing evidence indicates that OSA is correlated with cognitive dysfunction, excessive daytime sleepiness, difficulties in personal relationships, impaired work performance, anxiety, and an increased risk of automobile accidents [5]. Although much of the causal role and mechanisms are still poorly understood, activation of a series of neural, humoral, thrombotic, metabolic, and inflammatory disease mechanisms are hypothesized to be involved in the pathophysiologic mechanisms of OSA [2], [6], [7].

The etiology of OSA is complex; anatomic factors that promote pharyngeal narrowing, including large neck circumference, cervical soft tissue, vessels, and bony structures are recognized as the dominant etiology [8]–[10]. This view is supported by known effective treatments: continuous positive airway pressure (CPAP) and tonsillectomy, to a certain extent [11]. In addition to these anatomical factors, many risk factors are associated with OSA. The strongest risk factors for OSA are obesity, age and gender [12]. More than 60% of OSA patients can be classified as morbidly obese [13]. The prevalence of OSA in individuals >65 years of age is two- to three-fold higher than that among middle-aged (35–64 years) men and women [14]. The prevalence of OSA is higher in males than in females [15]. Environmental factors known to exacerbate OSA include alcohol ingestion, sedative use, sleep deprivation, and tobacco use [16]. While the correlation between risk factors and OSA is always confounded by obesity, the link between OSA and airway muscle dysfunction is certain. Airway muscle dysfunction might promote pharyngeal collapsibility by decreasing the caliber of the upper airway or by increasing the pressure surrounding the upper airway [17]. One study showed that throat muscles and tongues of OSA patients relax more than normal [18].

Muscles distributed in the tongue, throat, and upper airway are categorized as skeletal or smooth muscles [19]. Muscle cells contain actin and myosin protein filaments that slide past one another to achieve biological functions via contraction or relaxation [20]. The dystrophin-glycoprotein complex (DGC) is a cellular membrane anchor for cytoskeletal proteins that plays an important role in maintaining muscle cell function [20]. The DGC forms a link between the F-actin cytoskeleton and extracellular matrix; the sarcoglycan complex is a subcomplex within the DGC comprised of alpha-, beta-, gamma-, delta- and zeta-sarcoglycans [21]. The sarcoglycans are asparagine-linked glycosylated proteins with single transmembrane domains [21], [22]. Delta-sarcoglycan (SGCD) damage and gene defects are associated with hypertrophic cardiomyopathy [23], dilated cardiomyopathy [23], arrhythmogenic right ventricular cardiomyopathy and viral myocarditis [24], [25]. Sarcoglycan gene polymorphisms are associated with hypertrophic cardiomyopathy, myoclonus-dystonia syndrome and coronary spastic angina [26], [27].

Recently, although substantial progress has been made in identifying the genetic basis of some forms of sleep disorder, such as restless leg syndrome (RLS) and narcolepsy [28]–[30], the genetic basis of OSA remains unclear. Genetic factors clearly play a role in OSA, the genetic component of which has been estimated at 40% [31]. A study showed that over 43% of children with OSA have at least one relative with OSA symptoms [32]. Four genome-wide linkage analysis studies in European-Americans, African-Americans and Filipinos identified several regions of the genome with suggestive linkage to OSA [31], [33]–[35]. Commonly studied candidate genes in non-genome-wide association studies have focused on adrenergic receptors, angiotensin converting enzyme, leptin, serotonin receptors and transporters, endothelins, and apolipoproteins, and several positive associations have been reported [36]. A recent meta-analysis showed that the TNFA variant –308 A/G at rs1800629 was significantly associated with OSA under an additive model [37]. The possible link between SCGD gene SNPs and OSA has not yet been evaluated. Thus, in this study, five candidate SNPs in the SCGD gene were selected, and the genotype distributions were determined among 101 individuals without OSA and 97 OSA patients.

## Results

### Candidate SNPs from the SGCD gene

The SGCD gene is located at 5q33.2-33.3. As many as 9,386 SNPs exist in the SGCD gene database (http://www.genecards.org/cgi-bin/carddisp.pl?gene=SGCD), six of which have been well studied. As shown in Table 1, the genetic polymorphisms rs157350 [38], rs7715464 [39], rs32076 [40], rs13170573 [26], [27] and rs1835919 [41] are associated with brachial/hip circumference and paclitaxel sensitivity in the NCI60 cancer cell line, Ca2+ signaling in selenium resistance in the NCI60 cell line, coronary spasm in Japanese patients with hypertrophic cardiomyopathy, hypertrophic cardiomyopathy and norepinephrine secretion, respectively. For the abovementioned five SNPs, information regarding the location, global minor allele frequencies and allele frequencies in the Southern Han Chinese population is summarized in Table 1. One more SNP, a C→T substitution at nucleotide residue 84 (TAC→TAT) in exon 2, causing no amino acid alteration (Tyr→Tyr), reportedly associated with hypertrophic cardiomyopathy in Japanese and Mexican populations [42], [43], was not included in this study because we were unable to locate it in current SNP databases. Thus, five candidate SNPs (rs157350, rs7715464, rs32076, rs13170573 and rs1835919) of the SGCD gene were examined here.

**Table 1:** Candidate SNPs in the SGCD gene.

SNPID	Location	Allele	Global minorallelefrequency	SouthernHanChinese	Association	References
rs157350	Chr5:156,712,558	A/G	G = 0.0629/137	G = 0.9950	Brachial and hipcircumference	Croat Med J 2009,50: 7–16. Ref. 38
rs7715464	Chr5:156,382,042	A/G	A = 0.1272/277	A = 0.0850	Paclitaxelsensitivity inNCI60 cancercell lines	BMC Med Genomics 2011,4∶18. Ref. 39
rs32076	Chr5:156,684,212	C/T	T = 0.1791/389	T = 0.1650	Ca2+ signaling inselenium resistancein the NCI60cell line	PLoS ONE 5: e12601.Ref. 40
rs13170573	Chr5:156,327,1825′-untranslatedregion	G/C	G = 0.4747/1033	G = 0.7850	Coronary spasm inJapanese patientswith hypertrophiccardiomyopathy	Circ J 2007,71∶1263–7. Ref. 26
					Hypertrophiccardiomyopathy	Genet Test Mol Biomarkers 2012,16∶855–8. Ref. 27
rs1835919	Chr5:156,186,481intron 4	A/G	G = 0.2557/556	G = 0.6200	Norepinephrinesecretion	J. Neurochem 2013,127∶750–61. Ref. 41

All candidate SNPs included in this study met the following criteria: 1) reportedly associated with a certain phenotype; 2) valid in the NCBI database; and 3) have a definite location.

### Study population characteristics

Individuals with (n = 97) and without OSA (n = 101) were recruited into this study randomly. Age, body mass index (BMI) and waist circumference/hip circumference ratio (WHR) of the OSA group were significantly higher than that of the control group (Table 2). The proportions of individuals with triglyceride (TG) levels ≥1.7 mmol/L, low-density lipoprotein cholesterol (LDL-C) levels ≥3.33 mmol/L, hypertension, and current smokers were significantly higher in the OSA group than in the control group (Table 2). Conversely, the proportion of females was significantly lower in the OSA group than in the control group (Table 2). Our OSA population was representative of the characteristics that have been reported in most current studies [43].

**Table 2:** Baseline data of the study groups.

Variable	Without OSA	With OSA	*P*-value
	(n = 101)	(n = 97)	
Age, yr (range)	40 (31–53)	48 (32–55)	0.0321
Female, N (%)	40 (39.6)	21 (21.6)	0.0047
BMI (kg/m^2^)	23.7 (21.7–25.7)	25.4 (21.5–27.3)	0.0032
WHR	0.90 (0.85–0.94)	0.93 (0.86–0.95)	0.0124
TG ≥1.7 mmol/L, n (%)	22 (21.8)	35 (36.1)	0.0193
HDL-C <1.03 mmol/L, n (%)	37 (36.6)	40 (41.2)	0.3021
LDL-C ≥3.33 mmol/L, n (%)	19 (18.8)	32 (33.0)	0.0169
AHI	1.3 (0.4–2.8)	9.2 (6.9–11.7)	0.0015
Smoker, n (%)	21 (20.8)	37 (38.1)	0.0056
Drinker, n (%)	18 (17.8)	21 (21.6)	0.3091
Presence of hypertension, n (%)	11 (10.8)	22 (22.7)	0.0205
Presence of diabetes, n (%)	5 (4.9)	11 (11.3)	0.0819

Skewed data are presented as medians (interquartile range) and categorical data as numbers (percentage). Differences in baseline characteristics were determined using Student’s t-tests, Fisher’s exact tests or χ^2^ tests according to the data distribution characteristics.

Abbreviations: BMI, body mass index; WHR, waist circumference/hip circumference ratio; TG, triglyceride; HDL-C, high-density lipoprotein cholesterol; LDL-C, low-density lipoprotein cholesterol; apoE, apolipoprotein E; AHI, apnea-hypopnea index.

### SNP genotype frequencies in OSA patients and controls

To investigate any possible link between SCGD gene SNPs and OSA, the genotypes of all candidate SNPs were determined in OSA patients and controls. The Hardy-Weinberg equilibria (HWE) of all genotypes were evaluated using online software (http://www.oege.org/software/hwe-mr-calc.shtml). The allele frequencies of all SNPs in the OSA and control groups were in HWE (P>0.05), suggesting that the genotype frequencies in the OSA and control populations remained constant in terms of their genetic background (Table 3). The genotype frequencies of rs157350, rs7715464, rs32076 and rs1835919 were close to those reported in the Southern Han Chinese population by the 1000 Genomes Project (http://www.1000genomes.org/) (Table 1 and Table 3). Of the genotype frequencies for rs157350, rs7715464, rs32076 and rs1835919, no significant differences were observed between the OSA and control groups (Table 3). For rs13170573, the GG, GC and CC genotypes in the control versus OSA groups were 51.5% vs. 37.1%, 36.6% vs. 35.1%, and 11.9% vs. 27.8%, respectively (Table 3). Significantly fewer OSA patients possessed the GG genotype and significantly more possessed the CC genotype compared with controls (Table 3). In conclusion, rs13170573, but not rs157350, rs7715464, rs32076 or rs1835919, was significantly correlated with OSA in this study.

**Table 3:** Genotype frequencies in OSA patients and controls.

Genotype	Without OSA	With OSA	*P*-value
	(n = 101)	(n = 97)	
*rs157350*			
AA	53 (52.5%)	50 (51.6%)	1.0000
AG	40 (39.6%)	40 (41.2%)	0.8851
GG	8 (7.9%)	7 (7.2%)	1.0000
*P* for HWE	0.72	0.72	
*rs7715464*			
AA	10 (9.9%)	9 (9.3%)	1.0000
AG	50 (49.5%)	48 (49.5%)	1.0000
GG	41 (40.6%)	40 (41.2%)	1.0000
*P* for HWE	0.35	0.34	
*rs32076*			
CC	41 (40.6%)	40 (41.2%)	1.0000
CT	40 (39.6%)	38 (39.2%)	1.0000
TT	20 (19.8%)	19 (19.6%)	1.0000
*P* for HWE	0.6	0.61	
*rs13170573*			
GG	52 (51.5%)	36 (37.1%)	0.0463
GC	37 (36.6%)	34 (35.1%)	0.8825
CC	12 (11.9%)	27 (27.8%)	0.0069
*P* for HWE	0.7	0.55	
*rs1835919*			
AA	36 (35.6%)	35 (36.1%)	1.0000
AG	38 (37.6%)	36 (37.1%)	1.0000
GG	27 (26.7%)	26 (26.8%)	1.0000
*P* for HWE	0.54	0.55	

Genotype frequencies are shown as numbers and percentages. Hardy-Weinberg equilibrium (HWE) was examined using free online software (http://www.oege.org/software/hwe-mr-calc.shtml).

### rs13170573 is associated with OSA, as determined by multivariate logistic regression analysis

To further confirm the link between rs13170573 and OSA, multivariate logistic regression analysis was adopted to eliminate any influences caused by unknown confounders. The distributions of age, gender, BMI and WHR as well as the proportions of current smokers and of individuals with TG ≥1.7 mmol/L, LDL-C ≥3.33 mmol/L, and hypertension were significantly different between the OSA patients and controls in our preceding assessment. Thus, to evaluate any independent contribution of these factors on OSA, we employed multivariate logistic regression modeling and used all these variables together with rs13170573 genotypes as input. As shown in Table 4, we found that the CC genotype of rs13170573 was an independent risk factor for OSA with an odds ratio (OR) of 2.17 (95% confidence interval [CI]: 1.19–6.01) (Table 4). Other variables, such as age ≥50 years, male gender, BMI ≥25 kg/m^2^, LDL-C ≥3.33 mg/dL, smoking and hypertension, were also independent risk factors for OSA, with various ORs determined by multivariate logistic regression analysis (Table 4). In conclusion, the CC genotype of rs13170573 was an independent risk factor for OSA in our study.

**Table 4:** Factors associated with OSA in our multivariate analysis.

Factor	Category	OR	95% CI
rs13170573 genotypes	CC	2.17	1.19–6.01
	GC	0.57	–2.32–5.21
	GG	1.00	/
Age	≥50 years	1.99	1.11–5.32
Sex	Male	2.35	1.78–4.78
BMI	≥25 kg/m^2^	4.32	2.21–7.11
LDL-C	≥3.33 mg/dL	1.42	1.11–2.73
Smoker	Yes	1.32	1.03–2.78
Hypertension	Presence	1.12	1.01–4.21

LDL-C, low-density lipoprotein cholesterol; BMI, body mass index; OR, odds ratio; CI, confidence interval.

## Discussion

SGCD is essential for formation of the sarcoglycan protein complex, which is comprised of alpha-, beta-, gamma-, delta- and zeta-sarcoglycans [20]–[22]. The sarcoglycan protein complex is located in the membrane of muscle cells. It helps to maintain muscle cell structure by attaching to and stabilizing the dystrophin complex [20]–[22]. Mutations in the SGCD gene are associated with limb-girdle muscular dystrophy and dilated cardiomyopathy [23]–[25]. Limb-girdle muscular dystrophy is a group of related disorders characterized by muscle weakness, particularly in the hips, shoulders and limbs [44]. Thus far, at least 14 SGCD gene mutations have been identified in people with limb-girdle muscular dystrophy type 2F [45]. Dilated cardiomyopathy is a condition in which the heart becomes enlarged and cannot pump blood efficiently; a small number of individuals who develop dilated cardiomyopathy without skeletal muscle involvement have a mutation in one copy of the SGCD gene in each cell [23]. Fewer studies exist on SGCD genetic polymorphisms compared with mutations in the SGCD gene. However, sporadic studies have indicated that SNPs in the SGCD gene are associated with brachial and hip circumference, paclitaxel sensitivity in the NCI60 cancer cell line, Ca2+ signaling in selenium resistance in the NCI60 cell line, coronary spasm in Japanese patients with hypertrophic cardiomyopathy, hypertrophic cardiomyopathy and norepinephrine secretion [26], [27], [38]–[41]. These studies suggested that SGCD gene SNPs also play important roles in pathophysiology. In this study, five candidate SNPs were selected to evaluate any possible correlation between OSA and the SGCD gene. Our study showed that the CC genotype of rs13170573 is highly prevalent in OSA patients, and further multivariate logistic regression analysis demonstrated that the CC genotype is an independent risk factor for OSA, with an OR of 2.17 (95% CI: 1.19–6.01). Other SNPs, such as rs157350, rs7715464, rs32076 and rs1835919, showed no correlation with OSA.

The G allele frequencies of Global minor allele frequency and Southern Han Chinese are 47.5% and 78.5% respectively (Table 1). In our study, G allele frequencies in control and case are 69.8% and 54.6% respectively. The differences between our data and 1000 Genomes Project might due to sampling bias and population composition. The prevalences of the GC heterozygote of rs13170573 in OSA patients and controls were 35.1% and 36.6%, respectively, which suggested that only the CC homozygote was associated with OSA. Because of this, we did not calculate the single allele prevalences in our populations. The mechanism responsible for the correlation between rs13170573 and OSA is unknown; rs13170573 is located in the 5′-untranslated region of the SCGD gene, and thus any function it has should be dependent on transcriptional regulation. However, rare SNP studies demonstrated a simple correlation between a specific SNP and target gene regulation, suggesting that SNP phenotypic involvement is complex [46]. The CC genotype gene might alter the function of SCGD in muscles distributed in the respiratory tract. To evaluate muscle dysfunction in individuals possessing the CC genotype, a questionnaire that included questions regarding heart disease and myoclonus-dystonia syndrome was administered, and no complaints related to the above diseases were noted. Further physical examination also eliminated the existence of hypertrophic cardiomyopathy, dilated cardiomyopathy and muscle dysfunction. Available information regarding rs13170573 is limited. The C allele is a significant risk factor for coronary spasm in patients with hypertrophic cardiomyopathy in both Japanese and Mexican populations [26], [27]. Thus, the exact mechanism and contribution of the SGCD CC genotype in OSA need further study.

In this study, age, BMI and WHR were significantly higher in the OSA group than the control group. The proportions of current smokers and of individuals with TG ≥1.7 mmol/L, LDL-C ≥3.33 mmol/L, and hypertension were also significantly higher in the OSA group than the control group. The proportion of females was significantly lower in the OSA group than the control group. These results are consistent with many studies on OSA and suggest that our OSA population is representative [43].

Current thinking regarding SNP studies has entered a new phase involving genome-wide association studies (GWAS). The advantages of GWAS include a) coverage of the entire genome, which allows for hypothesis-free testing of all genes; b) data that can be used for quality control and estimation of population stratification; and c) consistent replication of the detected associations [37]. Nonetheless, GWAS have not yet been performed on OSA-related SNPs [37], because these studies still very time-consuming and costly. Because of this, candidate gene study is the best choice for many research groups. The limitations of this study included a lack of functional assessment of the rs13170573 genotypes and a small study population. Small study population makes us impossible to study the association between rs13170573 genotypes and severity of OSA by further stratification.

## Materials and Methods

### Patients

This study was conducted in accordance with the amended Declaration of Helsinki. The Review Board of the Minhang District Center Hospital approved the study protocol (reference number: SHMHCH 2008-0014). Written informed consent was obtained from all patients according to the guidelines of the Chinese National Ethics Regulation Committee; we explained the procedure to all patients and emphasized that their data would be used in this study. All patients were informed of their right to withdraw consent either personally or via kin, caretakers, or guardians.

An OSA patient referred for diagnosis was first screened by a single physician and next subjected to whole-night polysomnography. An abnormal breathing event was defined as complete cessation of airflow (apnea) for more than 10 s or a >50% reduction in respiratory airflow accompanied by a decrease in oxyhemoglobin saturation (SaO2) of >4% and/or an electroencephalographic arousal. OSA diagnosis was made according to “The International Classification of Sleep Disorders, Revised, Diagnostic and Coding Manual”, published by the American Academy of Sleep Medicine in association with the European Sleep Research Society, Japanese Society of Sleep Research and Latin American Sleep Society [1].

The questionnaire explored demographics, personal histories, and present illnesses. Anthropometric measurements were performed as reported previously by Sánchez-García et al. [47]. Blood pressure was measured according to the American Heart Association guidelines [48]. Diabetes was diagnosed using the American Diabetes Association (ADA) diagnostic criterion of a fasting glucose level ≥7.0 mmol/L [49].

### Candidate SNP selection and genotyping assay

Candidate SNPs were selected by the following strategies: 1) collection and screening of data from the GeneCards Human Gene Database (http://www.genecards.org/cgi-bin/carddisp.pl?gene=SGCD); 2) National Center for Biotechnology Information (NCBI) keyword search for the following research terms: (obstructive sleep apnea hypopnea or OSA or obstructive sleep apnea syndrome) and (delta-sarcoglycan or SCGD or sarcoglycan-delta) in combination with (gene or polymorphism or variants or alleles; 3) two medical doctors independently performed the data search, screening and extraction; disagreements regarding data screening and extraction were resolved by discussion; 4) all selected SNPs were confirmed in the databases maintained byNCBI SNP (http://www.ncbi.nlm.nih.gov/projects/SNP/) and the 1000 Genomes Project (http://www.1000genomes.org/). Finally, five SNPs (rs157350, rs7715464, rs32076, rs13170573 and rs1835919) along with their SNP ID, global minor allele frequency, and PubMed references were included in this study.

Genomic DNA was extracted from 5 mL peripheral blood from all samples using a QIAamp DNA Blood Midi Kit (Valencia, CA, USA). Allele-specific real-time polymerase chain reaction (RT-PCR) was used to examine the genotypes of all selected SNPs (TaqMan; SNP Genotyping Assays, Applied Biosystems, Foster City, CA, USA) on an ABI PRISM Genetic Analyzer 7900 (Applied Biosystems). Each SNP was confirmed using the NCBI SNP database and Applied Biosystems Celera Discovery System website with the following SNP IDs: rs13170573, rs157350, rs7715464, rs32076 and rs1835919.

### Statistical analyses

Skewed data are presented as medians (interquartile ranges) and categorical data as numbers (percentages). Differences in baseline characteristic were examined using Student’s t-tests, Fisher’s exact tests or χ2 tests according to the data distribution characteristics.

Hardy-Weinberg equilibria of the alleles at each individual locus were evaluated using free online software designed for detection of Hardy-Weinberg equilibrium (http://www.oege.org/software/hwe-mr-calc.shtml). Associations between the genotype at each locus and the presence of OSA were evaluated using χ^2^ tests. Possible confounding effects among the variables were adjusted using a multivariate logistic regression model, and ORs and 95% CIs were calculated. A P-value<0.05 was considered significant in the two-tailed tests [25]. All statistical analyses were performed using Stata/SE 12.0 for Windows (StataCorp LP, College Station, Texas).
